# Tethered Semiflexible Polymer under Large Amplitude Oscillatory Shear

**DOI:** 10.3390/polym11040737

**Published:** 2019-04-23

**Authors:** Antonio Lamura, Roland G. Winkler

**Affiliations:** 1 Istituto Applicazioni Calcolo, CNR, Via Amendola 122/D, 70126 Bari, Italy; 2 Theoretical Soft Matter and Biophysics, Institute for Advanced Simulation, Forschungszentrum Jülich, D-52425 Jülich, Germany

**Keywords:** mesoscale simulations, nonequilibrium simulations, LAOS, polymer dynamics

## Abstract

The properties of a semiflexible polymer with fixed ends exposed to oscillatory shear flow are investigated by simulations. The two-dimensionally confined polymer is modeled as a linear bead-spring chain, and the interaction with the fluid is described by the Brownian multiparticle collision dynamics approach. For small shear rates, the tethering of the ends leads to a more-or-less linear oscillatory response. However, at high shear rates, we found a strongly nonlinear reaction, with a polymer (partially) wrapped around the fixation points. This leads to an overall shrinkage of the polymer. Dynamically, the location probability of the polymer center-of-mass position is largest on a spatial curve resembling a limaçon, although with an inhomogeneous distribution. We found shear-induced modifications of the normal-mode correlation functions, with a frequency doubling at high shear rates. Interestingly, an even-odd asymmetry for the Cartesian components of the correlation functions appears, with rather similar spectra for odd *x*- and even *y*-modes and vice versa. Overall, our simulations yielded an intriguing nonlinear behavior of tethered semiflexible polymers under oscillatory shear flow.

## 1. Introduction

The structural and rheological properties of polymers are strongly affected by external fields, such as shear or extensional flows. As such, this is well established, and the external fields provide means to control the behavior of polymer solutions, melts, and networks [1,2,3,4,5]. Similarly, it has been shown that the macroscopic rheological behavior of a polymer solution, e.g., shear-rate dependent viscosities, normal-stress differences, and shear thinning, are tightly linked to the microscopic polymer conformational and dynamical properties [5,6,7,8,9,10,11]. Hence, insight into the behavior of individual polymers is fundamental in the strive to unravel the macroscopic nonequilibrium polymer properties.

Direct observation of the nonequilibrium properties of single molecules, also termed “molecular rheology” [5], in experiments [12,13,14,15,16,17,18,19] and simulations [10,13,20,21,22,23,24,25,26,27,28,29,30,31,32,33,34,35,36,37,38,39,40,41,42,43,44] has provided valuable visual illustrations of polymer conformations and has helped to characterize their nonequilibrium properties in terms of their deformation, orientation, relaxation dynamics, and rheology, both for free and tethered molecules in shear and extensional flow. These studies, however, typically probe the linear viscoelastic properties, which are usually insufficient to fully characterize the nonlinear aspects of polymers under flow.

In order to probe nonlinear properties of complex fluids, the large-amplitude oscillatory shear (LAOS) method was developed [19,45,46,47,48]. Here, in contrast to small-amplitude oscillatory shear, the stress response is typically no longer sinusoidal, but of rather complex shape. Such strong, time-dependent flows will affect the conformational properties of individual polymers to a yet unresolved extent. Studies on the dynamics of single polymers under large-amplitude oscillatory extensional flow yield qualitatively different stretching flow-rate curves (Lissajous curves) as a function of the extension rate and the oscillation frequency [19], and illustrate the complex interplay between time-dependent flows and polymer conformations. Here, further studies are desirable to resolve the time-dependent conformational properties of single polymers under large-amplitude oscillatory flows, aspects which, so far, have not been addressed by simulations.

In this article, we perform non-hydrodynamic, Brownian-type simulations of individual polymers exposed to large-amplitude oscillatory shear. The ends of the polymer are fixed and the polymer dynamics is constraint to the xy-plane of a Cartesian reference frame. We consider stiffer polymers only, with the persistence lengths $L_{p}/L=1/2$ and 2, respectively ($L_{p}$ is the persistence and *L* the polymer contour length). In any case, the polymers exhibit cyclic conformational modulations, specifically at higher Weissenberg numbers (Wi), which is the product of the applied shear rate and the longest polymer relaxation time. The tethering of the ends leads to a more-or-less linear oscillatory response at small Weissenberg numbers, where a polymer moves back and forth like grass swaying in the wind. With increasing Wi, polymers (partially) wrap around the fixation points and shrink. In general, the probability of the center-of-mass position is largest on a limaçon-type curve, as a consequence of the periodic excitation, however, with a non-uniform probability. Interestingly, the center-of-mass autocorrelation function normal to the line connecting the tethering points exhibits frequency doubling with respect to the imposed shear frequency, as a consequence of the non-crossability of the polymer and the tethering points. This reflects the symmetry breaking of the polymer dynamics during an oscillation cycle. Our findings illustrated the complex nonlinear interplay of polymer internal degrees of freedom and external periodic oscillations.

The rest of the paper is organized as follows. In Section 2, the polymer model and the coupling to the shear flow are described. Section 3 presents results for conformational and dynamical properties. Our findings are summarized in Section 4.

## 2. Model and Method

**Figure 1:**
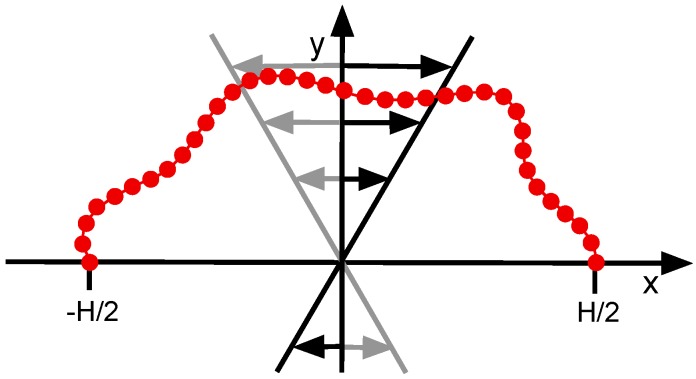
Sketch of the tethered bead-spring polymer exposed to oscillatory linear shear flow.

The two-dimensional linear polymer chain is composed of *N* beads of mass *M*, with its ends, $r_{1}$, $r_{N}$, tethered at $r_{1}={\left(-H/2,0\right)}^{T}$ and $r_{N}={\left(H/2,0\right)}^{T}$, respectively (cf. Figure 1). The contour length $L=\left(N-1\right)r_{0}$, where $r_{0}$ is the bond length, is fixed and L>H. The interactions between the beads are defined in terms of the potential $U=U_{bond}+U_{bend}+U_{ex}$, comprising bond, bending, and excluded-volume interactions. The bonds between consecutive beads are described by the harmonic potential:

(1) $$U_{bond}=\frac{\kappa_{h}}{2}\sum_{i=1}^{N-1}\left(\right|r_{i+1}-r_{i}{\left|-r_{0}\right)}^{2},$$

where $r_{i}$ is the position of bead *i* (i=1,…,N) and $\kappa_{h}$ is the elastic constant. Bending restrictions are captured by the potential:

(2) $$U_{bend}=\kappa \sum_{i=1}^{N-2}\left(1-\cos\varphi_{i}\right),$$

with κ the bending rigidity and $\varphi_{i}$ the angle between two consecutive bond vectors. In the limit κ→∞, the persistence length is given by $L_{p}=2\kappa r_{0}/k_{B}T$, with *T* the temperature and $k_{B}$ Boltzmann’s constant. Bead overlapping and bond crossings are prevented by the shifted and truncated Lennard–Jones potential:

(3) $$U_{ex}=4\epsilon \left[{\left(\frac{\sigma }{r}\right)}^{12}-{\left(\frac{\sigma }{r}\right)}^{6}+\frac{1}{4}\right]\Theta \left(2^{1/6}\sigma -r\right),$$

where *r* is the distance between two non-bonded beads, and Θ(r) is the Heaviside function; Θ(r)=0 for r<0 and Θ(r)=1 for r>0. The dynamics of the beads is described by Newton’s equations of motion, which are integrated by the velocity-Verlet algorithm with time step $\Delta t_{p}$ [49,50].

The polymer is coupled to a Brownian heat bath implemented via the Brownian (or random) multiparticle collision dynamics (B-MPC) approach [51,52,53]. Hence, no hydrodynamic effects are considered in the present work. MPC consist of streaming and collision steps, where collisions occur in regular time intervals of length Δt [52,54]. During streaming, the dynamics of the beads is described by Newton’s equations of motion. In the collision step, the velocities of the beads change in a stochastic manner. In B-MPC, the Brownian interaction of a bead with the surrounding fluid is implemented by a stochastic collision with a phantom particle, taking its momentum from the Maxwell–Boltzmann distribution of variance $Mk_{B}T$ and a mean, which is zero in absence of shear. In the presence of oscillatory shear in the xy-plan the mean momentum is $Mv_{i}^{s}\left(t\right)$, with the shear velocity:

(4) $$\begin{array}{c} v_{i}^{s}\left(t\right)={\left(\dot{\gamma }y_{i}\cos\left(\omega t\right),0\right)}^{T} \end{array}$$

at the time *t*; $\dot{\gamma }$ is the shear rate and ω the frequency (cf. Figure 1). The collision is implemented via the stochastic rotation dynamics variant of MPC [52,55,56]. Here, the relative velocity of a bead, with respect to the mean of the velocities of the bead and related phantom particle, is randomly rotated by angles ±α.

We choose the following parameters for the simulations: $\alpha =130^{\circ }$, M=5, $\Delta t=0.1t_{u}$, with the time unit $t_{u}=\sqrt{Mr_{0}^{2}/\left(5k_{B}T\right)}$, $\kappa_{h}r_{0}^{2}/\left(k_{B}T\right)=4\;\times \;10^{3}$, $\epsilon /(k_{B}T)=1$, $\sigma =r_{0}$, N=101, hence, the polymer length is $L=100r_{0}$, and $\Delta t_{p}=10^{-2}\Delta t$. The value of $\kappa_{h}$ guarantees that the polymer length is constant within 1% for all considered systems. Two bending rigidities are considered, corresponding to the persistence lengths $L_{p}/L=0.5$ and 2.

Simulations of free polymers yield the longest relaxations times $\tau_{r}/t_{u}=1.9\;\times \;10^{6}$ and $\tau_{r}/t_{u}=3.9\;\times \;10^{6}$ for the two stiffness values, determined from the end-to-end vector correlation function [11,40]. The strength of the shear flow is characterized by the Weissenberg number $Wi=\tau_{r}\dot{\gamma }$, for which the values Wi=10,25,50, and 100 are considered. The frequency ω is related to the Deborah number $De=\tau_{r}\omega$, where we set De=10.

The polymer, with H=L/5, is initialized with beads along a semi-elliptical contour with the major axis along the *y*-direction and minor axis along the *x*-axis. The polymers are equilibrated up to $5\;\times \;10^{6}t_{u}>\tau_{r}$, and data are collected up to the longest simulated time $t_{L}=10^{8}t_{u}$.

## 3. Results

**Figure 2:**
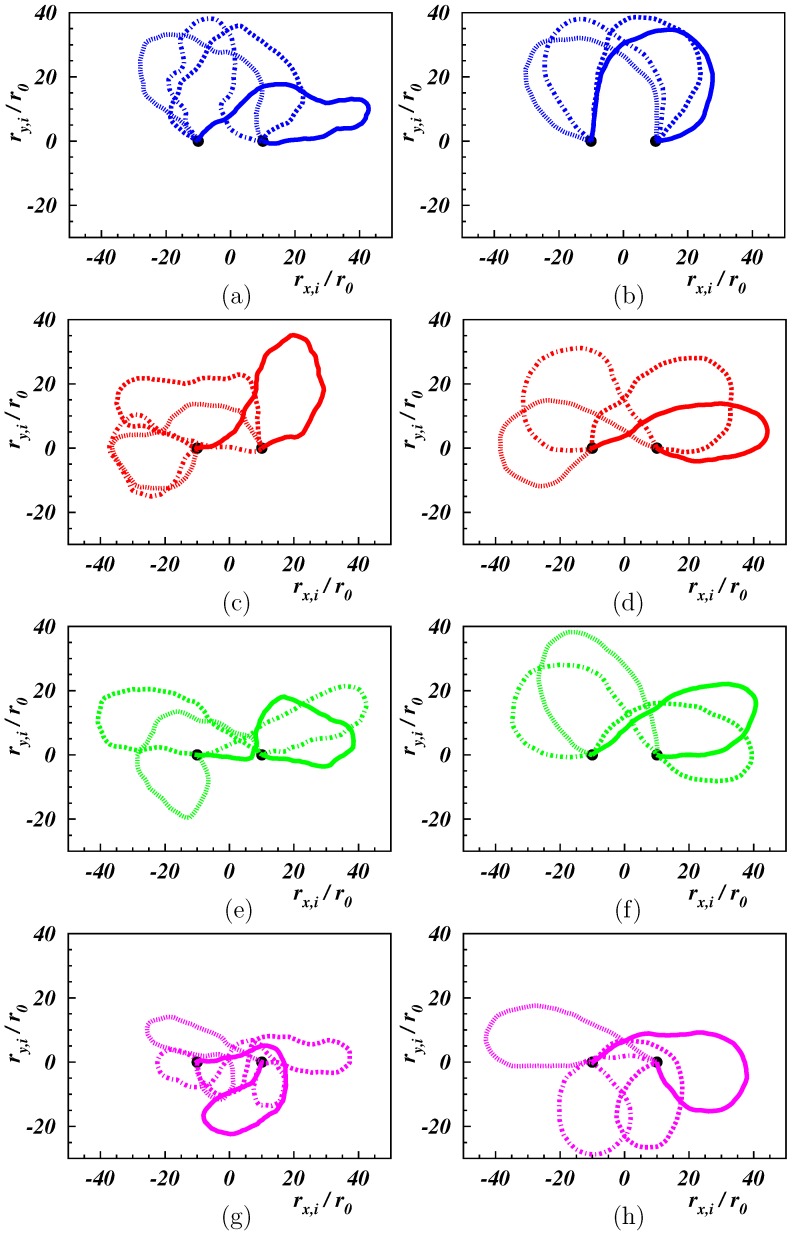
Conformations of the polymer at times t=0 (solid line), 0.5π/ω (dashed line), π/ω (dotted line), 1.5π/ω (dash-dotted line) after equilibration for $L_{p}/L=0.5$ (**left**), 2 (**right**), and Wi = 10, 25, 50, 100 (**from top to bottom**). Black dots denote the position of fixed beads.

The oscillatory flow dragged the beads along and, at least at small shear rates, the polymer moved back and forth like grass swaying in the wind. This is illustrated in Figure 2. The stiffer polymer ($L_{p}/L=2$) closely maintained its shape at low shear rates (Figure 2b). A flexible polymer was deformed more easily, but an in-phase oscillation was still present (Figure 2a). Larger Weissenberg numbers led to stronger conformational changes and larger bead displacements (Figure 2c–h).

### 3.1. Center-of-Mass Properties

**Figure 3:**
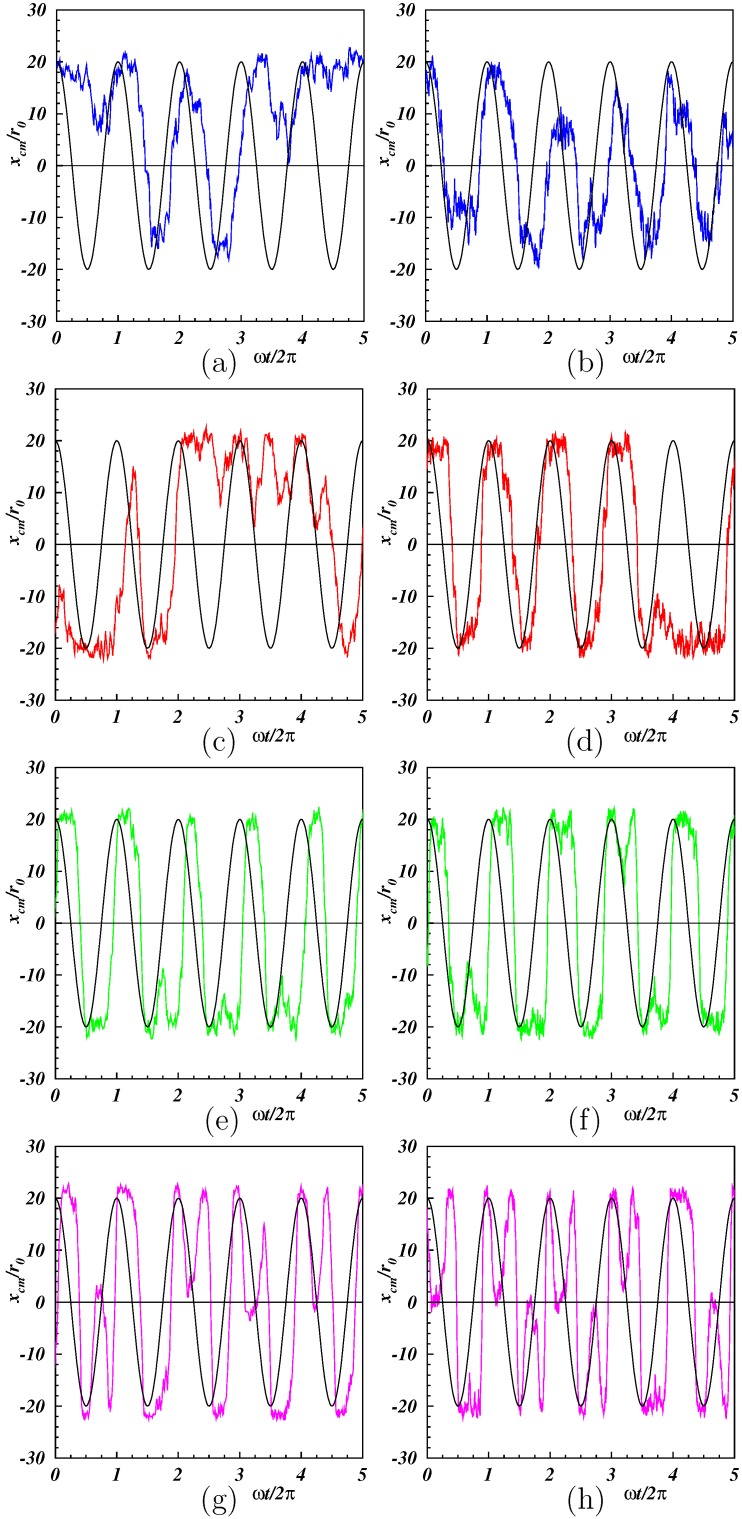
Time-dependence of the center-of-mass coordinate along the *x*-axis for $L_{p}/L=0.5$ (**left**) and 2 (**right**), and Wi = 10, 25, 50, 100 (**from top to bottom**). The black lines correspond to the externally applied shear flow with arbitrary amplitude.

Figure 3 shows the time-dependence of the *x*-coordinate of the center-of-mass position for various Weissenberg numbers. For smaller Wi≲25, entropic effects were strong, and $x_{cm}$ was only partially following the external flow. Perturbations were stronger when the polymer got trapped by the fixation points and some time was needed to disentangle it (cf. Supplementary Movies for $L_{p}/L=0.5,2$ and Wi=25). There were in-phase periods with a small phase shift, which were interrupted by time intervals with a center-of-mass motion decoupled from the flow. A stronger flow (Wi≈50) enhanced the in-phase periodic motion, but the center-of-mass dynamics was phase shifted and seemed to be no longer harmonic. The modulations of the approximately periodic peaks became more pronounced for Wi=100, and an original single peak split into two peaks, with the minimum of the second peak close to zero. Hence, $x_{cm}$ exhibited an approximate doubling of the frequency, an aspect which is more closely discussed in Section 3.2.2 in the context of normal modes. Overall, we found a highly nonlinear response of the polymer to the external excitation. This modified the polymer conformational and dynamical properties (cf. Supplementary Movies for $L_{p}/L=0.5,2$ and Wi=100).

**Figure 4:**
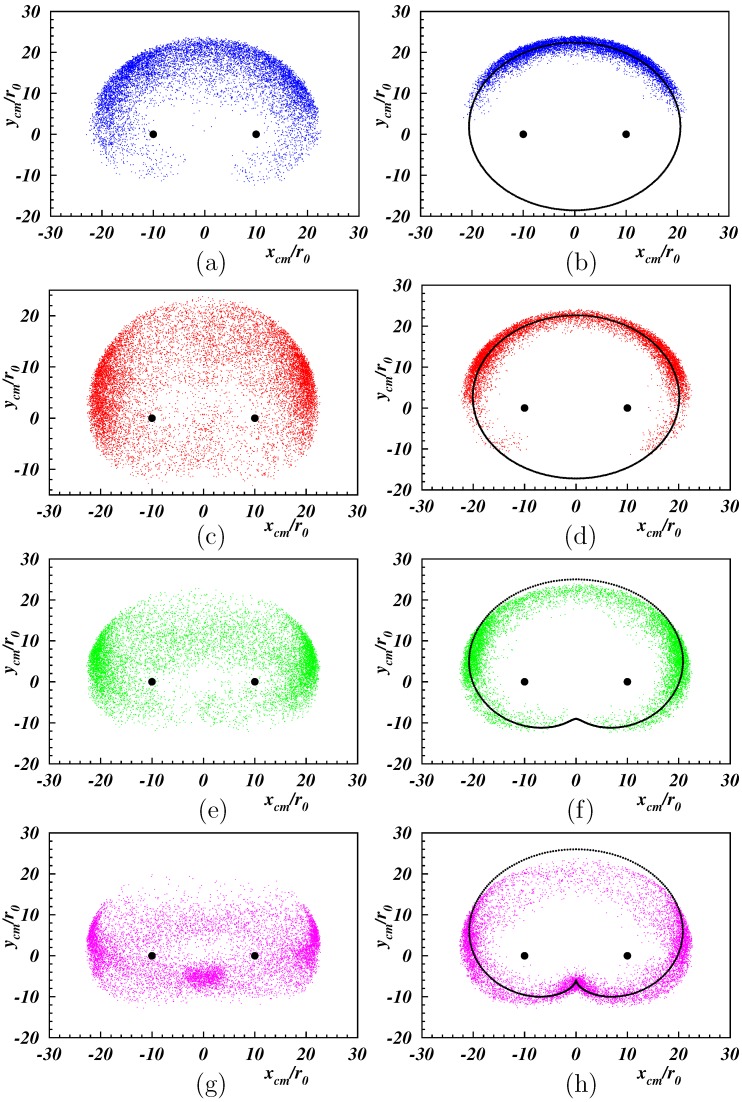
Probability distribution of the center-of-mass position of the tethered polymers for $L_{p}/L=0.5$ (**left**) and 2 (**right**), and Wi=10,25,50,100 (**from top to bottom**). Black dots indicate the position of fixed ends. The lines for $L_{p}/L=2$ (right) are limaons according to Equations (5) and (6) for the parameters: (**b**) $a/r_{0}=20.48$, $b/r_{0}=1.92$, $y_{0}=0$, (**d**) $a/r_{0}=19.91$, $b/r_{0}=2.72$, $y_{0}=0$, (**f**) $a/r_{0}=17$, $b/r_{0}=14$, $y_{0}/r_{0}=-6$, (**h**) $a/r_{0}=16$, $b/r_{0}=16$, $y_{0}/r_{0}=-6$.

Figure 4 depicts the probability distribution of the center-of-mass position for various Weissenberg numbers. The probability was high for positive $y_{cm}$-values, specifically at lower Wi. This was related to the chosen initial conditions, with a polymer always in the half-plane y>0. Evidently, the polymers were too stiff to restore isotropy normal to the shear-flow direction. The anisotropy was maintained at higher Wi. However, the most probable center-of-mass position shifted gradually to larger $|x_{cm}|$. At large Weissenberg numbers, the probability increased in the vicinity of $x_{cm}=0$. As illustrated in Figure 2g,h, the polymers were wrapped around the tethering points by the flow. Overall, the probability of the center-of-mass position was highest on a limaçon-type curve, however, with a non-uniform distribution. A limaçon is defined as a curve formed by the path of a point fixed to a circle, when that circle rolls around the outside of a circle of equal radius. In our case, the shear flow (partially) rotated (oscillated) the semiflexible polymer of more-or-less circular shape, which looked like rolling, and the center-of-mass followed a limaçon.

The polar coordinate representation of a limaçon is r=a+bcosθ, and the parameter representation for our reference frame is [57]:

(5) $$\begin{array}{cc} x= & \;r\cos\theta =\left[a+b\sin\theta \right]\cos\theta , \end{array}$$

(6) $$\begin{array}{cc} y= & \;y_{0}+r\sin\theta =y_{0}+\left[a+b\sin\theta \right]\sin\theta , \end{array}$$

with the off-set $y_{0}$. The fits of Equations (5) and (6) to our simulation results for $L_{p}/L=2$ are displayed in Figure 4. For Wi=10, the limaçon was hardly distinguishable from an ellipse. With increasing Weissenberg number, we approached a limaçon, and for Wi=100, the limaçon turned into a cardioid, where a=b. The limaçon curves for Wi=50, and especially Wi=100, did not fully agree with the simulation data for $y_{cm}/r_{0}≳10$. The reason was the conformational freedom of the polymer and shear-induced shape changes, implying changes in the radii of the rolling circles underlaying the mathematical limaçon construction. These changes were most pronounced while the polymer explored regions of high shear rate.

**Figure 5:**
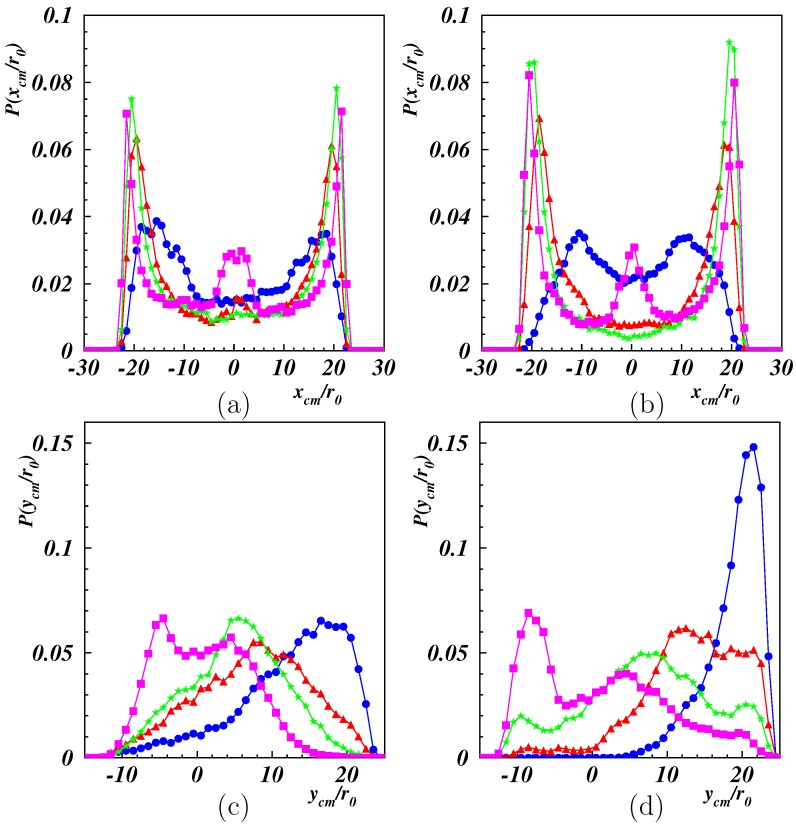
Probability distribution functions of the *x* (**top**) and *y* (**bottom**) coordinates of the polymer center of mass for $L_{p}/L=0.5$ (**left**), 2 (**right**) and Wi=10(•),25(▴),50(⋆),100(▪).

**Figure 6:**
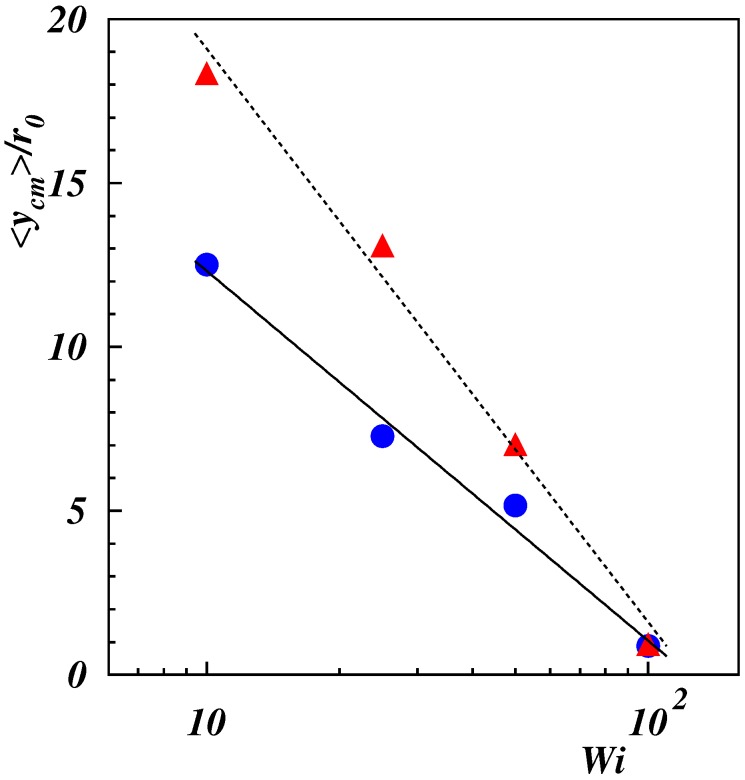
Average position along the *y*-direction of the polymer center of mass as a function of the Weissenberg number for $L_{p}/L=0.5$ (•), 2 (▴). Lines are the fits $<y_{cm}>/r_{0}∼\beta \ln Wi$ with β=−4.9±0.4 (full line) β=−7.6±0.6 (dashed line).

The distribution functions for the center-of-mass Cartesian coordinates of the polymers are displayed in Figure 5. The distribution function for $P(x_{cm})$ (Figure 5a,b) is symmetric with respect to the center between the tethered ends. At small shear, distinct “off-center” peaks were present as a consequence of the projection onto the *x*-axis. With the increasing Weissenberg number, the peaks became more pronounced and shift closer to the end positions. This is also reflected in the variance $\langle x_{cm}^{2}\rangle$, which increased with Wi. At high Weissenberg numbers, a peak appeared in the center, reflecting the polymer “wrapping” around the fixed ends. The asymmetry in the initial condition of $y_{cm}$ is also reflected in Figure 5c,d, with a pronounced peak at positive $y_{cm}$. The probability of smaller $y_{cm}$ increased with increasing Wi, and for Wi≳100, a peak appeared at $y_{cm}<0$, consistent with the high probability in the vicinity of $x_{cm}=0$ of Figure 4g,h. The snapshots in Figure 2g,h show stretching and alignment along the *x*-direction of the polymer-part between the fixed end point and the point where wrapping around the other fixed end appears. For such conformations, the major parts of the polymer were at y<0. The wrapping combined with the reversal of the polymer advective dynamics resulted in a slow lateral dynamics, resulting in a high probability with $y_{cm}<0$. Yet, the average $\langle y_{cm}\rangle$ was always positive, as shown in Figure 6, but decreased quickly with increasing Wi. A fit with the logarithmic Weissenberg-dependence $\langle y_{cm}\rangle =-\beta r_{0}\ln Wi$ yielded β=4.9 and 7.6 for $L_{p}/L=1/2$ and 2, respectively. Evidently, the drop is more pronounced for the stiffer polymer.

The stronger conformational changes with increasing strength of the shear flow imply a shrinkage of the polymer. The mean square radius of gyration shrinks by 10–15%, with respect to non-sheared conformation.

### 3.2. Conformational Properties

#### 3.2.1. Normal Mode Expansion

We studied the internal polymer conformational and dynamical properties via the mode amplitudes $A_{n}\left(t\right)={\left(A_{nx}\left(t\right),A_{ny}\left(t\right)\right)}^{T}$ of the eigenfunction expansion of a polymer with fixed ends [58],

(7) $$r_{i}=\frac{H(2i-N-1)}{2(N-1)}\hat{x}+2\sum_{n=1}^{N-1}A_{n}\sin\left(q_{n}\left[i-1\right]\right)\;,$$

with $\hat{x}$ the unit vector along the *x*-axis and the wave numbers $q_{n}=n\pi /\left(N-1\right)$ (n=1,…,N−1). The mode amplitudes are:

(8) $$\begin{array}{c} A_{n}=\frac{1}{N-1}\sum_{i=1}^{N-1}S_{i}\sin\left(q_{n}\left[i-1\right]\right), \end{array}$$

in terms of the bead positions $S_{ix}=r_{ix}-\left(2i-N-1\right)H/2\left(N-1\right)$ and $S_{iy}=r_{iy}$ (i=1,…,N).

**Figure 7:**
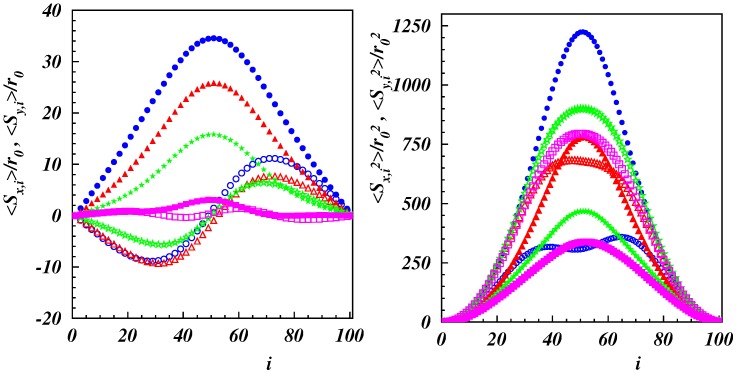
Mean (**left**) and mean square (**right**) values of the components of the displacement S as a function of the bead index, *i*, of the semiflexible polymer with $L_{p}/L=2$ for Wi=10(∘),25(▵),50(⋆),100(□). Open symbols indicate $S_{x}$ and filled symbols $S_{y}$.

The mean, 〈S〉, and mean square, $\langle S^{2}\rangle$, values of the components of S are displayed in Figure 7 for $L_{p}/L=2$. The behavior was qualitatively similar for $L_{p}/L=1/2$. The shape of $\langle S_{x}\rangle$ was centrosymmetric with respect the polymer center i=50. Any deviation was a consequence of statistical inaccuracy. The magnitude $\left|\langle S_{x}\rangle \right|$ decreased significantly with the increasing Weissenberg number, and the mean was close to zero for Wi=100. The component $\langle S_{y}\rangle$ was always positive and the largest amplitude was naturally in the polymer center. With increasing Wi, the amplitudes shrunk, and $\langle S_{y}\rangle$ was close to zero for all beads. The mean square values $\langle S_{y}^{2}\rangle$ (Figure 7, right) decreased with increasing Wi. However, $\langle S_{x}^{2}\rangle$ changed in a nonmonotonic manner, and the fluctuations were larger for Wi=50 than for Wi=100. This was a consequence of the symmetry-breaking shear flow with respect to the *x*-axis and the wrapping of the polymers around the tethering points.

**Figure 8:**
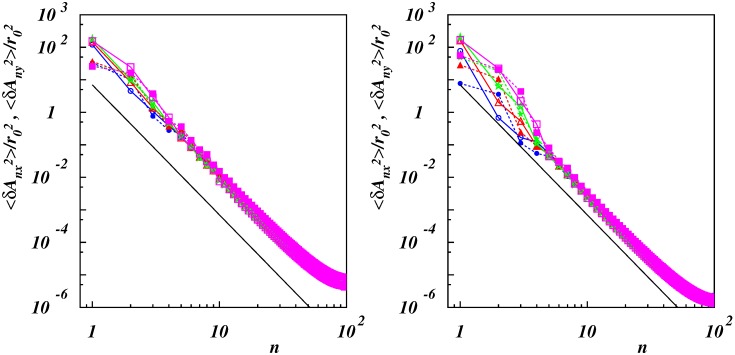
Variance of the mode components $A_{nx}$ (open symbols with solid lines) and $A_{ny}$ (solid symbols with dashed lines) as functions of mode number *n* for $L_{p}/L=0.5$ (**left**), 2 (**right**) and Wi=10(•),25(▴),50(⋆),100(▪). The slope of the solid lines is −4.

Figure 8 depicts the dependence of the variance of the mode amplitudes:

(9) $$\begin{array}{c} \langle \delta A_{n}^{2}\rangle =\langle {\left(A_{n}-\langle A_{n}\rangle \right)}^{2}\rangle , \end{array}$$

on the mode number for the two stiffness values and the various Weissenberg numbers. For small Wi, we obtained a $1/n^{4}$ dependence as characteristic for stiff polymers [59,60]. This even applied over a range of mode numbers 4<n≲40 at higher shear. However, the variances of the lower-mode amplitudes deviated from this dependence, with a weaker mode-number dependence for small *n*, and a rapid drop from mode n=3 to n=4. This reflected the large-scale conformational changes by the wrapping of the polymers.

#### 3.2.2. Dynamical Properties

**Figure 9:**
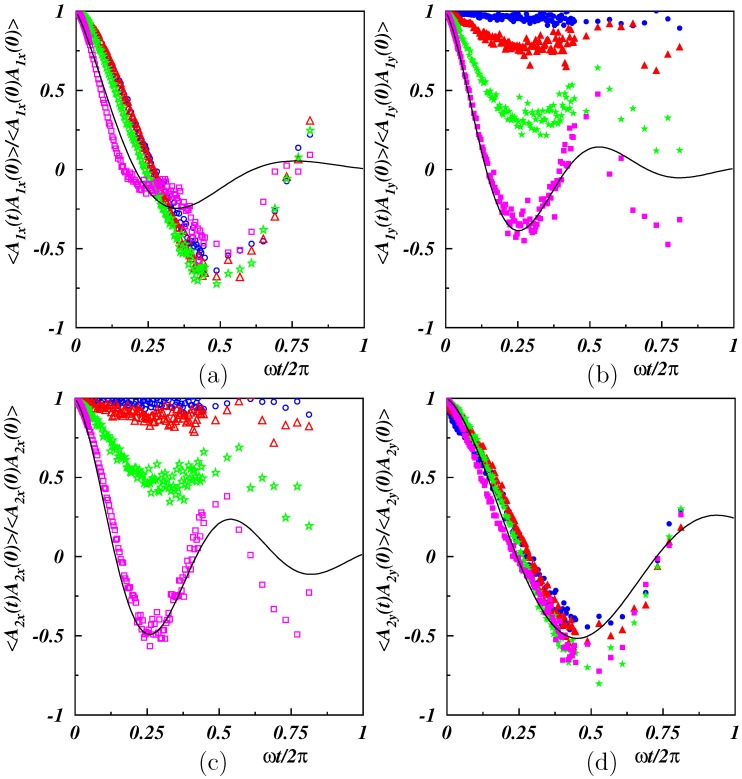
Autocorrelation function of the mode amplitudes for the modes n=1 (**top**) and n=2 (**bottom**) as a function of time along the *x*- (**left**) and *y*-direction (**right**). The polymer stiffness is $L_{p}/L=2$ and the Weissenberg numbers Wi=10(∘),25(▵),50(⋆),100(□). The black solid lines are fits of a damped sinusoidal oscillation.

Since the large-scale properties of the polymer were modified most by the shear flow, we more closely considered the dynamics of the mode amplitudes for the modes n=1 and 2 by the mode-autocorrelation function $C_{n}\left(t\right)=\langle A_{n}\left(t\right)\cdot A_{n}\left(0\right)\rangle$. Results for various Weissenberg numbers and the persistence length $L_{p}/L=2$ are presented in Figure 9. The simulation data were analyzed by fitting the exponentially damped periodic function:

(10) $$\begin{array}{c} F\left(t\right)=e^{-\gamma t/T_{0}}\cos\left(\Omega \omega t\right), \end{array}$$

where $T_{0}=2\pi /\omega$ is the period of the applied oscillation, γ characterizes the damping, and Ω accounts for variations of ω. The correlation $C_{1x}\left(t\right)=\langle A_{1x}\left(t\right)A_{1x}\left(0\right)\rangle$, in Figure 9a, decreased at short times and increased again for $t/T_{0}>1/2$. Fitting yielded a factor Ω≈1.2, i.e., $C_{1x}\left(t\right)$ followed roughly the shear flow. At high Weissenberg numbers, an additional weak modulation appeared, which, however, did not change the primary frequency. The decay of the correlation function $C_{1y}\left(t\right)=\langle A_{1y}\left(t\right)A_{1y}\left(0\right)\rangle$ showed a strong Weissenberg number dependence. As depicted in Figure 9b, $C_{1y}\left(t\right)$ depended only very weakly on time for Wi=10. With the increasing Weissenberg number, the decay rate, γ, increased, and the correlation function showed damped oscillations, which were most pronounced for Wi=100. Interestingly, the characteristic frequency was twice the externally applied frequency (Ω≈1.8). The correlation function $C_{2x}\left(t\right)$ exhibited a very similar time and Weissenberg number dependence as $C_{1y}\left(t\right)$ (cf. Figure 9c), in terms of drop at the various Wi, as well as the characteristic frequency. The correlation $C_{2y}\left(t\right)$ (cf. Figure 9d) was very similar to $C_{1x}\left(t\right)$, where the fitted expression closely followed the simulation data. Here, we found Ω≈1, i.e., the external frequency, but the decay was stronger with γ≈1.4. Moreover, within both sets of correlation functions, the exponential decay depended only weakly on the Weissenberg number. It was easily recognized that there was an odd-even asymmetry between the *x*- and *y*-correlation functions. This asymmetry was also obtained for higher mode numbers.

Theoretical models of flexible and semiflexible polymers predict an exponential decay of the normal-mode correlation functions of the form $e^{-t/\tau_{n}}$, where the $\tau_{n}$ are the relaxation times [6,61]. In the presence of external fields, e.g., shear flow, the time dependence is modified and the normal-mode correlation functions do not necessarily decay exponentially anymore [11], but the exponential factor is still determined by the relaxation times. We did find an initial exponential decay, but no clear mode-number dependence. Figure 8 shows a deviation of the smaller mode numbers from the dependence $\tau_{n}∼1/n^{4}$, but the relaxation times of the modes n=1 and 2 were still different. This is not reflected in Figure 9, where the decay of $\langle A_{1x}\left(t\right)A_{1x}\left(0\right)\rangle$ and $\langle A_{2y}\left(t\right)A_{2y}\left(0\right)\rangle$ differs by approximately a factor of 2.5, which is roughly the ratio of the mode numbers. Hence, the oscillatory flow field strongly affected the relaxation times, at least at higher Weissenberg numbers. This reflects and is consistent with a rather nonlinear response of the polymer with respect to the external periodic excitation.

**Figure 10:**
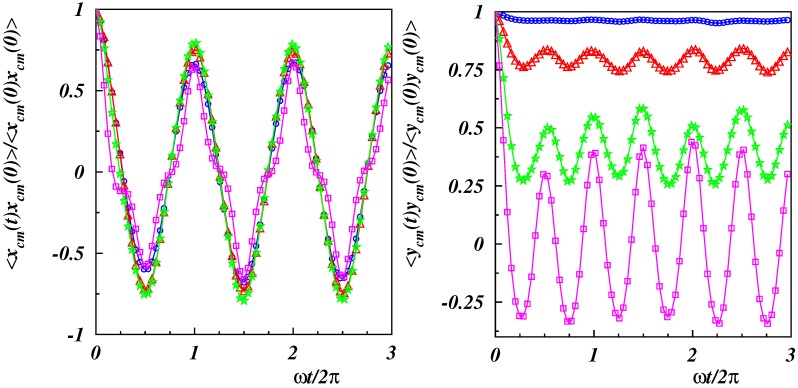
Autocorrelation function of center-of-mass Cartesian coordinates as a function of time along the *x*- (**left**) and *y*-direction (**right**). The polymer stiffness is $L_{p}/L=2$ and the Weissenberg numbers Wi=10(∘),25(▵),50(⋆),100(□).

The autocorrelation functions for the center-of-mass Cartesian coordinates displayed in Figure 10 help to understand the appearance of the frequency doubling. Evidently, both Cartesian components exhibited a periodic motion, with typically larger amplitudes for higher Weissenberg numbers. Clearly, the *x*-component shows the same frequency as the applied flow, with some modulations appearing for the highest value of Wi. In contrast, the *y*-component revealed frequency doubling for Wi≳25. The Supplementary Movies illustrate the different dynamical features of $x_{cm}$ and $y_{cm}$. In one period, the *x*-position of the center of mass, let us say, moved from the maximum positive value to the minimum value (half period) and, by the oscillation, back again to the maximum *x*-value. The oscillation was similar to a complete “rotation” (period), and no difference between oscillation and rotation was visible. The behavior of the amplitude of $y_{cm}$ was different. While in a cycle, $x_{cm}$ decreased, $y_{cm}$ increased first, reached its maximum for $x_{cm}=0$, and decreased then again. Instead of being able to complete a full cycle, the oscillation and the tethering constrained $y_{cm}$ to move back along a similar path as in the first half of the period, i.e., $\langle y_{cm}\left(t+T_{0}/2\right)\rangle$ increased, reached again a maximum and decreased then again to a value close to the initial value. Hence, $y_{cm}$ exhibited two maxima during a period, whereas $x_{cm}$ exhibited only one. As a consequence, the correlation function $\langle y_{cm}\left(t\right)y_{cm}\left(0\right)\rangle$ showed twice the frequency than the respective correlation function of the *x*-coordinate. The primary reason was the self-avoidance by the tethering points, which forced a reversal of the dynamics along the *y*-direction.

## 4. Summary and Conclusions

We have analyzed the nonequilibrium properties of semiflexible polymers confined in two dimensions with tethered ends exposed to oscillatory shear. The applied Brownian multiparticle collision dynamics algorithm neglected hydrodynamic interactions. It coupled the polymer dynamics in a stochastic (Brownian) manner to the local flow field and allowed for fluctuations, since the average flow field was imposed only.

For small shear rates, low Weissenberg numbers (Wi≲10), the tethering of the ends led to a more-or-less linear oscillatory response, where the polymers moved back and forth like grass swaying in the wind. With increasing Wi, the polymers (partially) wrapped around the fixation points and a more complex, nonlinear response emerged. In fact, the wrapping significantly changed the polymer conformations, and the overall size, measured by the radius of gyration, shrunk. Dynamically, the probability of the polymer center-of-mass position was largest on a spatial curve resembling a limaçon, although the distribution was inhomogeneous. Of course, this was a consequence of tethering and periodic excitation of the polymer, moving it cyclically back and forth, which led to an approximate rolling motion of the center-of-mass, the origin of the limaçon.

At high Weissenberg numbers, we found shear-induced modifications of the mode-spectrum of the mode-amplitude correlation functions. Since we considered semiflexible polymers only, the mode spectrum exhibited the characteristic $1/n^{4}$ dependence for low Wi. At high Wi, the lower modes, specifically the modes n⩽3, showed a weaker *n* dependence. This is reflected in a particular dynamical behavior, where a frequency doubling appeared for the normal-mode amplitudes along the *x*- and *y*-direction. However, the doubling appeared alternating for even and odd modes—the correlation function $\langle A_{1y}\left(t\right)A_{1y}\left(0\right)\rangle$ shows twice the frequency of $\langle A_{1x}\left(t\right)A_{1x}\left(0\right)\rangle$, whereas $\langle A_{2x}\left(t\right)A_{2x}\left(0\right)\rangle$ is similar to $\langle A_{1y}\left(t\right)A_{1y}\left(0\right)\rangle$. The frequency doubling is also reflected in the autocorrelation function of the *y*-coordinate of the center-of-mass position. It was a consequence of the hindered and truncated “rotational” motion of the polymer by the tethering points.

In the current simulations, hydrodynamic interactions (HI) have been neglected. Such interactions are of major importance for flexible polymers, since they significantly change the relaxation time spectrum, but are less relevant for semiflexible polymers, where they provide approximately logarithmic corrections only [62]. Hence, we expected only minor differences between the presented results for free-draining polymers and simulations, accounting for HI as long as the polymer motion follows instantaneously the fluid flow.

Our studies revealed an intriguing behavior of tethered polymers under oscillatory flow, which affects their macroscopic rheological behavior. Specifically, the overall shrinkage of the polymer reduced the viscosity. The consequences of the frequency doubling on macroscopic properties need to be further analyzed.
